# Metabolite profiles and the risk of metabolic syndrome in early childhood: a case-control study

**DOI:** 10.1186/s12916-021-02162-7

**Published:** 2021-11-26

**Authors:** Sandi M. Azab, Russell J. de Souza, Amel Lamri, Meera Shanmuganathan, Zachary Kroezen, Karleen M. Schulze, Dipika Desai, Natalie C. Williams, Katherine M. Morrison, Stephanie A. Atkinson, Koon K. Teo, Philip Britz-McKibbin, Sonia S. Anand

**Affiliations:** 1 Department of Medicine, McMaster University, Hamilton, ON Canada; 2 Department of Health Research Methods, Evidence, and Impact, Faculty of Health Sciences, McMaster University, Hamilton, ON Canada; 3 Centre for Metabolism, Obesity and Diabetes Research, McMaster University, Hamilton, ON Canada; 4 Population Health Research Institute, Hamilton, ON Canada; 5 Department of Chemistry and Chemical Biology, McMaster University, Hamilton, ON Canada; 6 Department of Pediatrics, McMaster University, Hamilton, ON Canada

**Keywords:** Metabolic syndrome, Cardiometabolic risk factors, Early childhood, Metabolomics, Continuous risk score, Tyrosine and alanine, Gluconeogenesis, Amino acids metabolism, Fatty acids metabolism

## Abstract

**Background:**

Defining the metabolic syndrome (MetS) in children remains challenging. Furthermore, a dichotomous MetS diagnosis can limit the power to study associations. We sought to characterize the serum metabolite signature of the MetS in early childhood using high-throughput metabolomic technologies that allow comprehensive profiling of metabolic status from a biospecimen.

**Methods:**

In the Family Atherosclerosis Monitoring In earLY life (FAMILY) prospective birth cohort study, we selected 228 cases of MetS and 228 matched controls among children age 5 years. In addition, a continuous MetS risk score was calculated for all 456 participants. Comprehensive metabolite profiling was performed on fasting serum samples using multisegment injection-capillary electrophoresis-mass spectrometry. Multivariable regression models were applied to test metabolite associations with MetS adjusting for covariates of screen time, diet quality, physical activity, night sleep, socioeconomic status, age, and sex.

**Results:**

Compared to controls, thirteen serum metabolites were identified in MetS cases when using multivariable regression models, and using the quantitative MetS score, an additional eight metabolites were identified. These included metabolites associated with gluconeogenesis (glucose (odds ratio (OR) 1.55 [95% CI 1.25–1.93]) and glutamine/glutamate ratio (OR 0.82 [95% CI 0.67–1.00])) and the alanine-glucose cycle (alanine (OR 1.41 [95% CI 1.16–1.73])), amino acids metabolism (tyrosine (OR 1.33 [95% CI 1.10–1.63]), threonine (OR 1.24 [95% CI 1.02–1.51]), monomethylarginine (OR 1.33 [95% CI 1.09–1.64]) and lysine (OR 1.23 [95% CI 1.01–1.50])), tryptophan metabolism (tryptophan (OR 0.78 [95% CI 0.64–0.95])), and fatty acids metabolism (carnitine (OR 1.24 [95% CI 1.02–1.51])). The quantitative MetS risk score was more powerful than the dichotomous outcome in consistently detecting this metabolite signature.

**Conclusions:**

A distinct metabolite signature of pediatric MetS is detectable in children as young as 5 years old and may improve risk assessment at early stages of development.

**Supplementary Information:**

The online version contains supplementary material available at 10.1186/s12916-021-02162-7.

## Background

Obesity and related metabolic sequelae such as type 2 diabetes are increasingly prevalent among children in high-income countries [1, 2]. The metabolic syndrome (MetS) is a cluster of risk factors including abdominal adiposity, elevated blood pressure, dyslipidemia, and dysglycemia and is associated with a 2.25-fold increased risk of cardiovascular disease in adults [3, 4]. No clear international consensus exists to define pediatric MetS due to variation in defining the metabolic components and differences in risk factor thresholds [5]. Thus, the prevalence of MetS in children and obese youth can vary from 0.4 to 5.5% and from 6 to 39%, respectively, depending on the adopted definition [5, 6]. Furthermore, it is equally unclear in children whether the MetS is a distinct condition that carries a higher risk than that of its individual components [7, 8]. For example, a dichotomous outcome for the complex MetS aggregate can underestimate risk and limit the power to detect an association [7, 9]. On the other hand, use of a continuous MetS risk score provides a useful alternative and is a better reflection of the physiological continuum between a healthy and an unhealthy metabolic profile [6, 10]. This holds true especially when tracking MetS in early childhood.

Metabolomics is the comprehensive characterization of small molecules in a biospecimen using state-of-the-art instrumentation based on mass spectrometry (MS) or nuclear magnetic resonance (NMR) [11, 12]. It offers the potential for biomarker discovery with insights into disease pathophysiology by capturing complex and dynamic interactions between genetics, post-translational modifications, gut microbiome activity, and environmental or dietary exposures [13, 14]. The metabolome reflects endogenous compounds (e.g., amino acids, lipids, and sugars), exogenous compounds derived from the diet or the environment (e.g., polyphenols and phthalates), and metabolites produced through gut microbial metabolism (e.g., trimethylamine-*N*-oxide or TMAO) [15].

Few studies have investigated pediatric metabolic disorders using metabolomics and have primarily focused on single risk factors, in particular obesity and insulin resistance [16–20]. Only three metabolomics studies derived an internal (*study-specific*) continuous MetS *z*-score, and all were conducted in adolescents and older children (10 years and up) [21–23]. In this case-control study of 456 Canadian children age 5 years, we investigated the metabolomic signature which characterizes MetS and its component traits.

## Methods

### Study participants

This study was conducted among children from the Family Atherosclerosis Monitoring In earLY life (FAMILY) study [24]. FAMILY is a prospective birth cohort study involving 857 predominantly white families that include 901 children recruited from Hamilton, Ontario, between 2002 and 2009 with a 10-year follow-up. Of the 676 children who completed a follow-up 5-year visit, 557 had complete anthropometric and clinical data and complete records of maternal gestational diabetes mellitus (GDM). Fasting serum specimens were available for 491 children who were considered for this study (consort diagram Additional file 1: Fig. S1).

### Biospecimen and clinical data collection

Data collection occurred at the 5-year follow-up visit through questionnaires, physical measurements, and laboratory analysis of biospecimens as published previously [25]. Fasting blood samples were collected and serum was fractionated within 2 h from collection according to standard protocols, stored at − 80 °C and shipped on dry ice [25]. Cholesterol (LDL, HDL, and total cholesterol), triglycerides, and glucose from the fasting serum samples were analyzed at the McMaster Children’s Hospital and Hamilton Health Sciences Central Research Laboratories following standardized analysis protocols [24, 26].

### MetS in children: MetS case selection

Cohort-specific percentiles were calculated for waist circumference, systolic blood pressure, and serum fasting glucose using the most complete data sets for each variable from the total number of children who attended the 5-year visit. Standardized BMI-for-age *z*-scores were derived based on WHO child growth standards [27]. Sex-specific 90th percentile thresholds for waist circumference, blood pressure, and glucose were 57.0 cm, 112 mm Hg, and 5.0 mmol/L in females and 55.4 cm, 110.3 mm Hg, and 5.1 mmol/L in males, respectively. The 50th percentile threshold for waist circumference was 51.1 cm in girls and 51.2 cm in boys. Children were classified as having a higher risk of MetS and referred to as cases if they had one or more of the following abnormalities: (1) z-BMI ≥ 75th percentile and waist circumference ≥ median, (2) z-BMI ≥ 75th percentile and systolic blood pressure ≥ 90th percentile, (3) fasting serum glucose ≥ 90th percentile, and (4) maternal GDM diagnosis. Two hundred twenty-eight children who met these criteria were classified as “MetS cases” and were age- and sex-matched 1:1 to 228 controls (Additional file 1: Fig. S1).

### IDEFICS MetS risk score derivation

Among these 456 children, we derived a continuous MetS risk score based on percentile curves created for 18,745 children aged 2.0–10.9 years of the European IDEFICS (Identification and prevention of Dietary-and lifestyle-induced health EFfects in children and infantS) cohort as reference [6, 28]. The MetS score was calculated summing sex- and age-specific *z*-scores according to the following formula by Ahrens et al. [6]: IDEFICS MetS score = *z*_waist circumference_ + (*z*_systolic blood pressure_ + *z*_diastolic blood pressure_)/2 + (*z*_triglycerides_ − *z*_HDL_)/2 + *z*_fasting glucose_. For more details on the IDEFICS MetS definition and score derivation, refer to Additional file 2: Supplemental Methods.

### Serum metabolome analysis

A validated, high-throughput method based on multisegment injection-capillary electrophoresis-mass spectrometry (MSI-CE-MS) [29, 30] was used for the analysis of polar ionic metabolites consistently measured in serum filtrate samples on an Agilent 6230 time-of-flight MS with a coaxial sheath liquid Jetstream electrospray ion source equipped to an Agilent G7100A CE (Agilent Technologies Inc., Mississauga, ON, Canada). This multiplexed separation platform takes advantage of a serial sample injection format of 13 samples [31, 32] within a single CE run including a pooled quality control (QC) sample prepared by combining equal aliquots of serum samples from all study participants (*n* = 456) for rigorous QC and data filtering procedures. Serum sample pre-treatment and data acquisition have been previously described in detail [30]. An iterative data workflow based on multiplexed injections pattern was used to reject spurious signals, redundant peaks, and background ions when performing nontargeted metabolomics based on analysis of a pooled serum sample that also served as QC for assessing technical precision [30, 31]. Fifty-eight serum metabolites were consistently measured in over 75% of the samples and satisfied QC criteria of a variance under 30% when using MSI-CE-MS under two configurations with positive and negative-ion mode detection. Fifty-two metabolites were unambiguously identified (level 1) and subsequently quantified using a calibration curve, where ion responses were normalized to a single internal standard. Six unknown serum metabolites were annotated based on their characteristic accurate mass and relative migration time under positive or negative ion mode. 2-hydroxybutyric and 3-hydroxybutyric acids were not baseline resolved and were reported as the sum of both isomers (which led to a higher variance of 40%). Metabolite combinations and ratios frequently used in the literature were derived for branched chain amino acids as the sum of leucine, isoleucine and valine, and glutamine/glutamate ratio. Any non-detects were replaced by a value that was 1/5 the detection limit, set to the smallest value in the data set. Finally, a robust QC-based batch correction algorithm was used to adjust for long-term signal drift of the mass spectrometer [33]. This stringent data pre-processing approach ensured that only fully authenticated serum metabolites were included to reduce false discoveries. Principal component analysis was used for data visualization (i.e., data trends/outlier detection) of the serum metabolome using MetaboAnalyst 4.0 [34]. Additional file 3: Table S1 lists the identification of all detected metabolites based on m/z:RMT:mode, molecular formula, the mean concentration measured, the % CV, and the % of data completeness for each metabolite.

### Covariates

#### Dietary assessment

Detailed dietary information at 5 years of age was collected using a self-administered semiquantitative food frequency questionnaire completed by the mother for her child [24]. Detailed methods of the derivation of an overall diet quality score have been described previously [35, 36]. Briefly, 102 food items were harmonized to create 36 common food groups. A diet quality score was then calculated as the sum of daily servings of “healthy” foods (fermented dairy, fish and seafood, vegetables, legumes, fruits, nuts, and whole grains) minus the sum of daily servings of “unhealthy foods” (processed meats, refined grains, french fries, snacks, sweets, and sweet drinks).

#### Physical activity

Based on the Habitual Activity Estimation Scale (HAES) [24], the mother estimated the percentage of time her child spent at different periods of the day (between wake time and breakfast, breakfast and lunch, lunch and supper, and supper and bedtime) in each of the following activity levels: (a) *inactive*: sleeping, lying down, resting, napping; (b) *somewhat inactive*: sitting, reading, watching TV, playing video games, playing quiet games, or activities which are mostly done sitting down; (c) *somewhat active*: walking, climbing stairs, household chores; and (d) *active*: running, jumping, skipping, bicycling, skating, swimming, skipping, and games that require a great deal of these types of movements. Based on total awake time and after subtracting time spent at each meal, activity % was transformed into min/day for each of the four activity levels. The final physical activity variable was derived as a weighted sum of the *somewhat active* and *active* categories in minutes/day.

#### Other covariates

Sleep time, screen time, and socioeconomic status were also evaluated for association with MetS. Sleep at night was calculated in hours/day. Screen time was expressed in hours/day of combined television and computer use averaged between weekend and weekday. Children with low (< 1 h), medium (1 to < 2 h), and high (≥ 2 h) screen exposures were given a score of 1, 2, and 3, respectively, to investigate whether there is a trend with increasing screen exposure above the thresholds recommended by the American Association of Pediatrics and the Canadian sedentary behavior guidelines at age 5 [37]. Socioeconomic status was measured using a previously validated “Social Disadvantage Index” comprised of a 6-level score based on income, marital status, and employment [38]. There was no adjustment for ethnicity as all participants were white Caucasian.

### Statistical analysis

Descriptive statistics are presented for the overall sample (*n* = 456) and by controls (*n* = 228) and cases (*n* = 228). Continuous variables including anthropometrics, blood pressure, and laboratory measurement are presented as mean and standard deviation, and categorical variables are presented as counts and percentages. Most metabolites were not normally distributed; thus, all metabolites were natural-log transformed and auto-scaled (mean-centered then standardized) for further analysis. Significant metabolite associations with MetS were tested using multivariable logistic regression as follows. To build this model, we identified a set of covariates known from the literature to be important predictors of MetS, which included diet quality, physical activity, time spent outside, screen time, night sleep, breastfeeding, socioeconomic status, and maternal education. Among the 456 study participants, all covariates were available for 433 children (95%). As a first step, we tested each of these in a series of univariate regression models against MetS; screen time was the only covariate which was associated with MetS with a *p =* 0.036, so this was retained as a covariate in all future models. Next, we entered all the previously identified predictors into a forward stepwise selection model to test if their addition on top of screen time explained more variance than screen time alone; from this procedure, screen time and diet quality were retained. Our final maximally adjusted analysis kept these two variables along with physical activity, night sleep, social disadvantage index, and child’s age and sex to improve direct comparability with previous work in the field. Additionally, we tested the MetS-metabolite associations in intermediate adjusted models to confirm the consistency of results (Additional file 4: Table S2). Next, to assess the association between the metabolites and the continuous MetS score, we used multivariable linear regression adjusting for the same covariates as in the maximally adjusted logistic model described above. Statistical significance for the multivariable logistic regression analyses was set at *p* < 0.05. Bonferroni correction for multiple hypothesis testing was considered for the multivariable linear regression model with a *p* value threshold < 0.0008 (0.05/60 tests). All analyses, tables and graphs were completed in R (v3.6.3; R Foundation: A Language and Environment for Statistical Computing). Finally, a pathway analysis was conducted by significant metabolites’ names to understand which pathways the identified metabolites may affect using MetaboAnalyst 5.0 pathway analysis tool [34] in conjunction with a literature review relevant to MetS and related conditions (Additional file 5: Table S3).

## Results

### Participants characteristics

**Fig. 1:**
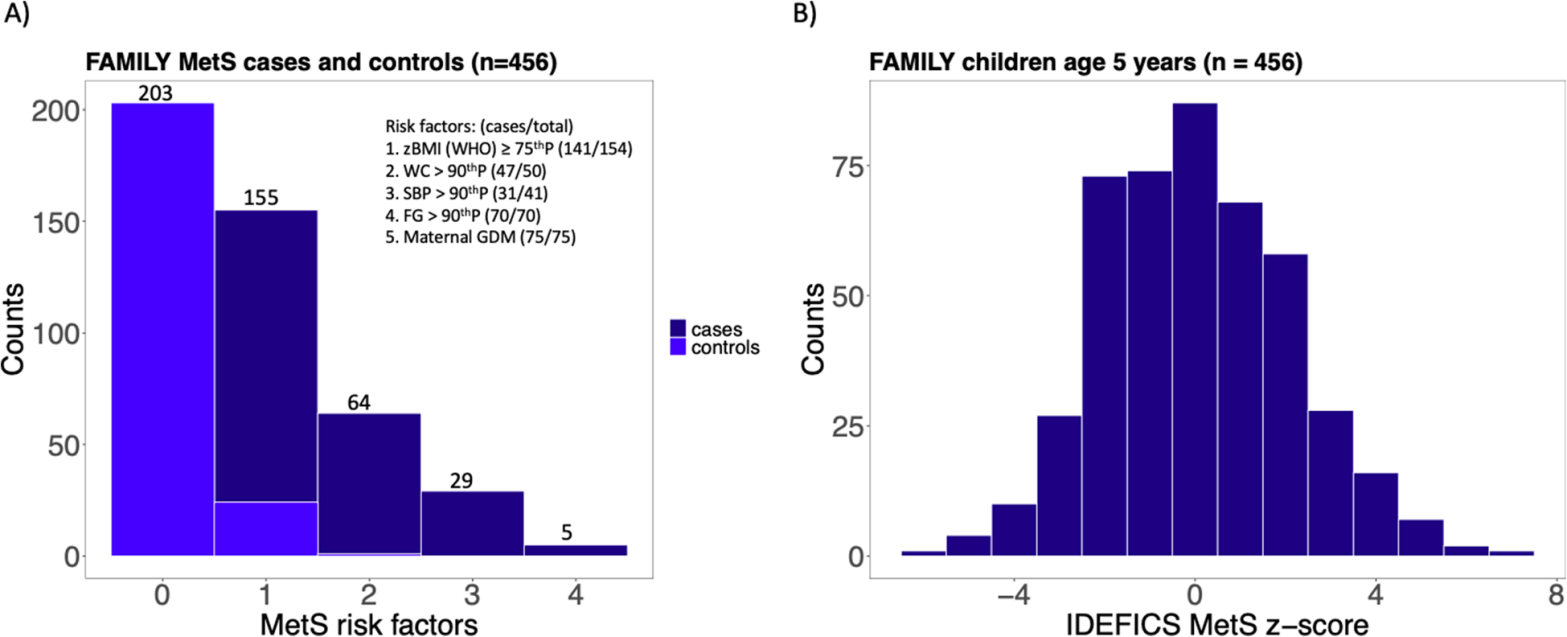
Participants’ metabolic syndrome (MetS) risk factors and MetS risk score distribution. **A** Bar graph of the clustering of MetS risk factors in FAMILY cases compared to controls. 57.4% of MetS cases had one risk factor, 27.6% had two risk factors, and 15% had more than two risk factors. **B** Histogram showing the distribution of the IDEFICS MetS score in study participants at age 5 years. The mean IDEFICS MetS z-score for all 456 was 0.05 and overall, the study participants at 5 years followed a normal distribution of the IDEFICS MetS score around a mean of zero. IDEFICS MetS score = *z*_waist circumference_ + (*z*_systolic blood pressure_ + *z*_diastolic blood pressure_)/2 + (*z*_triglycerides_ − *z*_HDL_)/2 + *z*_fasting glucose_

Characteristics of the study population included in this analysis (*n* = 456) are shown in Table 1. The mean age of the participants was 5.15 years; 50.4% were girls. Various measures for adiposity and values for glucose, systolic blood pressure, and diastolic blood pressure were higher in cases than in controls whereas serum lipids were not (Table 1). 57.4% of MetS cases had one risk factor, 27.6% had two risk factors, and 15% had more than two risk factors (Fig. 1A and Additional file 6: Fig. S2).

**Table 1:** Characteristics of MetS cases and controls^a^ in FAMILY

Characteristic	MetS case^b^ (*n* = 228)	MetS control (*n* = 228)	Overall (*n* = 456)
Age (years)	5.14 (0.28)	5.16 (0.29)	5.15 (0.29)
Sex (female)	115 (50.4%)	115 (50.4%)	230 (50.4%)
Daily night sleep (hours)	10.88 (0.65)	10.93 (0.61)	10.90 (0.63)
Physical activity (weighted daily minutes)	237 (71)	227 (71)	232 (71)
Diet quality Index	− 0.82 (3.4)	− 1.37 (3.45)	− 1.1 (3.43)
Social disadvantage index			
Low (0–1)	164 (73.5%)	163 (75.1%)	327 (74.0%)
Moderate (2–3)	43 (19.3%)	48 (22.1%)	91 (21.0%)
High (4–5)	16 (7.2%)	6 (2.8%)	22 (5.0%)
Screen time exposure			
Low exposure (< 2 h)	53 (23.3%)*	71 (31.8%)	124 (28.0%)
High exposure (≥ 2 h)	174 (76.7%)*	152 (68.2%)	326 (72.0%)
Systolic blood pressure (mm Hg)	101.30 (8.31)***	97.37 (8.03)	99.33 (8.40)
Diastolic blood pressure (mm Hg)	61.18 (5.44)***	59.25 (5.42)	60.21 (5.51)
Total cholesterol (mmol/L)	4.13 (0.65)	4.07 (0.72)	4.10 (0.69)
HDL cholesterol (mmol/L)	1.43 (0.32)*	1.37 (0.31)	1.40 (0.31)
LDL cholesterol (mmol/L)	2.38 (0.61)	2.37 (0.63)	2.37 (0.62)
Triglycerides (mmol/L)	0.71 (0.31)	0.69 (0.29)	0.70 (0.30)
Fasting blood glucose (mmol/L)	4.75 (0.38)***	4.52 (0.31)	4.63 (0.37)
BMI (kg/m^2^)	16.66 (1.80)***	15.13 (0.90)	15.89 (1.62)
BMI-for-age *z*-score (WHO)	0.84 (1.01)***	− 0.12 (0.64)	0.36 (0.97)
Waist circumference (cm)	53.43 (4.47)***	49.97 (2.59)	51.70 (4.04)
Waist circumference-to-height	0.48 (0.04)***	0.45 (0.02)	0.47 (0.03)
Sum of skinfolds (mm)	20.50 (6.78)***	16.80 (4.14)	18.63 (5.91)
z-BMI ≥ 75th percentile AND waist circumference ≥ 90th percentile	45 (19.7%)	0 (0%)	45 (9.9%)
z-BMI ≥75th percentile AND median ≤ waist circumference < 90th percentile	89 (39.0%)	0 (0%)	89 (19.5%)
z-BMI ≥75th percentile AND systolic blood pressure ≥ 90th percentile	22 (9.7%)	0 (0%)	22 (4.8%)
Fasting glucose ≥ 90th percentile	70 (30.7%)	0 (0%)	70 (15.4%)
Maternal GDM	75 (32.9%)	0 (0%)	75 (16.4%)

Values are presented as mean (SD) or *n* (%). ^a^Age- and sex-matched 1:1 with control children. ^b^Statistical comparisons between MetS cases and controls assuming equal (*t* test) or unequal variance (Welch’s *t* test) were performed as appropriate for continuous variables; Fisher exact tests were used for categorical variables. *p < 0.05, **p < 0.01, ***p < 0.001

### Prevalence of MetS and the IDEFICS MetS risk score

According to the IDEFICS criteria the proportion of children at 5 years with MetS was 2.9% (95% CI 2.0–3.7%) [6]. The IDEFICS definition is met when the value of *three or more* risk factors exceeds the 90th percentile for waist circumference, systolic OR diastolic blood pressure, HOMA-insulin resistance OR fasting glucose, and triglycerides OR is lower than the 10th percentile for HDL cholesterol. The prevalence of 2.9% would require a very large sample size to conduct association studies and therefore we employed a case-control design for the primary analysis.

The age- and sex-specific continuous MetS risk score and its individual components are presented in Table 2. The mean IDEFICS MetS score for cases was 1.00 and for controls was − 0.90, which further validated our selection criteria of cases and controls. Overall, the study participants at five years followed a normal distribution of the IDEFICS MetS score around a mean of zero (Fig. 1B).

**Table 2:** IDEFICS MetS score characteristics of MetS cases and control children in FAMILY

Characteristic	MetS cases (*n* = 228)	MetS control (*n* = 228)	Overall (*n* = 456)
IDEFICS MetS score	1.00 (2.06)	− 0.90 (1.58)	0.05 (2.07)
z-waist circumference	0.50 (1.29)	− 0.6 (0.94)	− 0.05 (1.25)
z-systolic blood pressure	0.38 (0.95)	− 0.08 (0.97)	0.15 (0.99)
z-diastolic blood pressure	− 0.19 (0.87)	− 0.50 (0.89)	− 0.34 (0.89)
z-triglycerides	0.51 (0.73)	0.47 (0.72)	0.49 (0.72)
z-HDL-cholesterol	0.41 (0.88)	0.22 (0.89)	0.32 (0.89)
z-lipids (triglycerides-HDL)	0.05 (0.66)	0.12 (0.66)	0.08 (0.66)
z-glucose	0.36 (0.83)	− 0.13 (0.67)	0.11 (0.79)

Values are presented as mean (SD)

### Metabolite profiling in 5-year-old children

#### Comparison of serum metabolite profiles in MetS cases and MetS controls

Comparing the metabolite profiling of fasting serum samples in 228 MetS children cases to age- and sex-matched 228 controls, we identified 12 serum metabolites that were significantly associated with MetS (*p* < 0.05) in the maximally adjusted multivariable model, in addition to glucose as anticipated (Table 3). For alanine, tyrosine, and monomethylarginine, each SD increment in log marker was associated with 33–41% increased odds of MetS, whereas tryptophan and glutamine/glutamate ratio were associated with 20% decreased odds. An unidentified unknown anion (*m/z* 248.0711) was associated with decreased odds of MetS. These results were consistent among all other intermediate adjusted models as shown in Additional file 4: Table S2.

**Table 3:** Relation of serum metabolome profiles to risk of MetS in young children with maximal adjustment for covariates

Metabolite	OR	95% CI	*p*-
Glucose	1.55	(1.25–1.96)	0.0001
Alanine	1.42	(1.16–1.75)	6.8e−4
Tyrosine	1.34	(1.10–1.64)	0.004
Monomethylarginine	1.35	(1.09–1.66)	0.005
Tryptophan	0.79	(0.65–0.96)	0.017
Choline	0.79	(0.64–0.96)	0.023
Deoxy carnitine	1.25	(1.03–1.52)	0.028
Carnitine	1.24	(1.03–1.52)	0.029
Threonine	1.24	(1.02–1.52)	0.031
Lysine	1.23	(1.01–1.50)	0.043
Arginine	1.22	(1.00–1.49)	0.048
Unknown 248.0711	0.82	(0.67–1.00)	0.049
Glutamine/glutamate	0.83	(0.68–1.00)	0.053

OR, odds ratio; CI, confidence intervals; *p*-, *p* value for statistical significance

Adjustment for screen time exposure, diet quality score, physical activity, sleep time, maternal social disadvantage index, child’s sex and age (23 (5.0%) missing values; final *n* numbers: 216 controls and 217 cases)

#### Association of serum metabolite profiles with the continuous MetS score

The continuous IDEFICS MetS score was used as an outcome variable because it would increase statistical power over use of the dichotomous outcome in detecting metabolomic associations. In the maximally adjusted model among 433 children, the IDEFICS MetS *z*-score was directly correlated with glucose, alanine, tyrosine, threonine, carnitine, monomethylarginine, and lysine and inversely correlated with tryptophan, and glutamine/glutamate ratio. These nine associations were consistent with the case to control results, and six of them satisfied Bonferroni correction for multiple hypothesis testing (*p* < 0.0008) (Table 4). Eight novel metabolites which were not identified in the case-control analysis were identified to be associated with the MetS *z*-score (*p* < 0.05) as summarized in Table 4. The metabolomics results in children age 5 years between the continuous and binary outcomes were largely in agreement although as definitions differed, so too did the metabolites which passed the statistical significance thresholds.

**Table 4:** Associations of fasting serum metabolites to the IDEFICS MetS score in young children

	Adjustment for screen time exposure, diet quality score, physical activity, sleep time, and maternal social disadvantage index				Adjustment for screen time exposure, diet quality score, physical activity, sleep time, maternal social disadvantage index, sex and age			
Metabolite	Estimate	SE	*z* value	*p-*	Estimate	SE	*z* value	*p-*
Glucose^a^	0.64	0.095	6.70	3.9e−11^b^	0.65	0.095	6.80	3.50e−11^b^
Alanine^a^	0.56	0.097	5.76	1.3e−10^b^	0.55	0.098	5.62	3.41e−08^b^
Tyrosine^a^	0.42	0.098	4.25	2.1e−5^b^	0.41	0.099	4.16	3.87e−05^b^
Threonine^a^	0.35	0.099	3.57	4.2e−4^b^	0.34	0.10	3.42	0.001
Carnitine^a^	0.41	0.097	4.17	3.4e−5^b^	0.41	0.098	4.19	3.35e−05^b^
Tryptophan^a^	− 0.36	0.099	− 3.59	4.6e−4^b^	− 0.36	0.099	− 3.62	3.2e−4^b^
Acetylcarnitine	− 0.42	0.098	− 4.26	3.1e−5^b^	− 0.42	0.098	− 4.23	2.92e−05^b^
Hydroxybutyric acids	− 0.38	0.098	− 3.89	2.1e−4^b^	− 0.39	0.098	− 3.96	8.64e−05^b^
Methionine	0.34	0.099	3.44	0.001	0.32	0.10	3.24	0.001
Proline	0.32	0.10	3.15	0.002	0.31	0.10	3.06	0.002
Arginine	0.30	0.10	2.95	0.003	0.29	0.10	2.86	0.004
Monomethylarginine^a^	0.29	0.10	2.79	0.006	0.30	0.10	2.85	0.005
Glutamic acid	0.27	0.10	2.72	0.007	0.26	0.10	2.57	0.011
Unknown 129.066	− 0.27	0.10	− 2.62	0.009	− 0.26	0.10	− 2.57	0.011
Glutamine/glutamate^a^	− 0.25	0.10	− 2.53	0.012	− 0.25	0.10	− 2.44	0.015
Lysine^a^	0.23	0.10	2.26	0.024	0.22	0.10	2.12	0.035
3-methyl-2-oxovaleric acid	− 0.22	0.099	− 2.26	0.024	− 0.22	0.099	− 2.24	0.026

^a^Consistent with adjusted logistic regression analysis

^b^*p* value passed Bonferroni correction *p* < 0.0008

## Discussion

We identified a unique panel of fasting serum metabolites associated with MetS in young children using nontargeted metabolomics by MSI-CE-MS. Circulating metabolites related to gluconeogenesis (glucose, alanine, and glutamine/glutamate ratio), amino acids metabolism (tyrosine, threonine, monomethylarginine, tryptophan and lysine), and fatty acids metabolism (carnitine) were associated with pediatric MetS consistently using both definitions after adjusting for age, sex, screen time, diet quality, physical activity, night sleep and socioeconomic status. Additionally, acetylcarnitine, hydroxybutyric acids, methionine, proline, arginine, 3-methyl-2-oxovaleric acid, and an unknown cation (m/z 129.066) had significant associations with the continuous MetS score.

In this well-characterized cohort of healthy 5-year-old children, our study design focused on the presence of any cardiometabolic risk-enhancing factors in early childhood, which considered both waist circumference and BMI together for adiposity, systolic blood pressure, hyperglycemia, and maternal GDM. Exposure to the latter increases the long-term risk of obesity and glucose intolerance in the offspring [39]. Yet, pediatric MetS remains ambiguously defined in the literature as there are no reference values in children, and studies can be biased to one risk factor over the other [5]. Consequently, our primary analysis was complemented by calculating the continuous MetS score that included diastolic blood pressure and lipid measurements. This score was adapted from external thresholds and is thus more generalizable. The consistency between both methods of analyses addresses an ongoing debate as to whether MetS is equal to, or more than, the sum of its constituent components. We hereby report a unique metabolite signature associated with MetS as an aggregation of risk factors.

Both tyrosine and alanine were previously found as potential early markers for the onset of insulin resistance and were positively associated with adiposity in children of various ethnicities [19, 20, 22, 23, 40]. In fact, Hellmuth et al. postulated that tyrosine elevations in obese children precede elevations in branched chain amino acids [41]. Insulin increases the activity of tyrosine aminotransferase, an enzyme that catalyzes tyrosine transamination and while in insulin resistance, circulating insulin may maintain adequate glucose metabolism, tyrosine breakdown may be affected [41]. Würtz et al. reported association of circulating tyrosine levels with intimal medial thickness—a surrogate for subclinical atherosclerosis—in young adults [42]. Alanine, on the other hand, is a central energy-related metabolite related to gluconeogenesis and the alanine-glucose cycle, which allows for recycling of hepatic glucose [43]. Our findings support the role of tyrosine and alanine in metabolic perturbations early in life before perturbations take place for other aromatic or branched chain amino acids, as well-established for adult diabetes [11]. Branched chain amino acids were not reflective of early stages of MetS in our cohort of generally healthy children in their fifth year of life. Tryptophan, an essential aromatic amino acid, was inversely correlated with MetS in young children. This could be attributed to low-grade inflammation which enhances tryptophan degradation to kynurenine [44]. However, kynurenine and other tryptophan metabolites [45] (e.g., serotonin, xanthurenic acid) were below method detection limits in our work.

Glutamine/glutamate ratio has been frequently reported in relation to metabolic abnormalities [46] and was inversely associated with pediatric MetS in our analysis. It has been hypothesized that glutamate increases glucagon release as well as transamination of pyruvate to alanine, promoting gluconeogenesis [45, 46]. Glutamine, in contrast, has been shown to reduce inflammation, inversely associate with diabetes risk and overall associate with metabolic wellness [46, 47]. Elevated serum carnitine, which functions as a shuttle carrier for fatty acids into muscle and liver cells for mitochondrial β-oxidation [48], may indicate reduced lipid oxidation in MetS cases. This hypothesis is supported by reduced serum acetylcarnitine and hydroxybutyric acids, which are products of lipolysis [49]. This is opposite to metabolic alterations observed in adults and could suggest an initial stage of maladaptation in early childhood of hyperinsulinemia and/or reduced ketogenesis [18]. More research is needed to investigate the role of diet and physical exercise in modifying the MetS metabolite signature. For instance, carnitine mostly comes from dietary meat consumption such as beef and lamb and can be considered as a non-quantitative marker of foods of animal origin, although it is also influenced by other factors such as age and health status [50].

Our study has several strengths—our metabolomics analysis in 456 children is the largest to date to investigate the MetS cluster as opposed to its individual components and the only study in early childhood as early as age 5 years. To the best of our knowledge, it is the first to derive the continuous MetS risk score drawn from the large-scale IDEFICS population. In addition, we examined possible covariates and adjusted the metabolite to MetS outcome for all significant covariates the most significant of which was screen time exposure which deserves further attention as a key driver of pediatric MetS, especially now amidst COVID-19 pandemic policies [51]. Next, we applied a correction to account for the number of statistical tests performed, to avoid false positive associations. Several limitations of the study also deserve mention. Although we used a nontargeted approach for unknown discovery, our metabolome coverage was limited to polar ionic metabolites excluding important lipophilic metabolites such as fatty acids [52] and phospholipids. Lipid profile was not incorporated in our MetS selection criteria, but this limitation was overcome by including triglycerides and HDL-cholesterol measurements in the continuous score. Physical activity was measured by maternal assessments on behalf of their children and not through objective accelerometer measurements and did not specifically measure extracurricular activities. Lastly, study participants were generally healthy and MetS cases were defined by presence of any single MetS component trait. A cohort with a higher prevalence of overweight children and related MetS traits may yield other novel metabolomic findings. Thus, a limitation of this study may be our definition of MetS cases, and the establishment of a formal MetS definition for children aged 5 years should still be pursued.

## Conclusion

In conclusion, from a panel of more than 60 fasting serum metabolites, a strong metabolic signature emerged with putative biomarkers of MetS risk in early childhood. Given the alarming rise in obesity among children, inadequate physical activity, intensified screen exposures, and lockdowns in an age group designed to be mostly active, early prognosis of MetS is extremely valuable.

## Supplementary Information

**Additional file 1: Fig. S1.** [consort chart for MetS case selection]

**Additional file 2.** Supplemental Methods [IDEFICS MetS definitions and MSI-CE-MS data processing]

**Additional file 3: Table S1.** [Serum metabolites characteristics]

**Additional file 4: Table S2.** [Unadjusted and intermediate adjusted models]

**Additional file 5: Table S3.** [Pathway analysis]

**Additional file 6: Fig. S2.** [Clustering of MetS cases]

## Data Availability

The datasets used and/or analyzed during the current study are available from the corresponding author on reasonable request.

## Abbreviations

MetS: Metabolic syndrome
FAMILY: Family Atherosclerosis Monitoring In earLY life
OR: Odds ratio
MS: Mass spectrometry
NMR: Nuclear magnetic resonance
GDM: Gestational diabetes mellitus
IDEFICS: Identification and prevention of Dietary-and lifestyle-induced health EFfects in children and infantS
MSI-CE-MS: Multisegment injection-capillary electrophoresis-mass spectrometry
QC: Quality control
HAES: Habitual Activity Estimation Scale
