# Supplementary material for: Metabolite profiles and the risk of metabolic syndrome in early childhood: a case-control study
Source: BMC Med. 2021 Nov 26;19:292. doi: 10.1186/s12916-021-02162-7 (PMC8616718; doi:10.1186/s12916-021-02162-7)
Supplement: Supplementary file 1 — Additional file 1: Fig. S1. [consort chart for MetS case selection] [file 12916_2021_2162_MOESM1_ESM.docx]

**Additional file 1:** **Figure S1: Consort chart for MetS case selection in FAMILY children age five years**

Used for percentiles cutoffs calculation:

(WHO) zBMI-for-age for BMI percentiles & sex-specific cohort percentiles for waist circumference, systolic blood pressure and serum fasting glucose

MetS cases:

1) (WHO) z-BMI ≥ 75^th^P & WC ≥ median (58.7%)

2) (WHO) z-BMI ≥ 75^th^P & SBP ≥ 90^th^P (9.7%)

3) FG > 90^th^P (30.7%)

4) maternal GDM (32.9%)

9 cases out of 237 were unmatched and excluded (all girls)

## Enrolment

## Inclusion

## Eligibility

## Availability

Only include singleton pregnancies

(n = 491)

Met Syndrome controls

(n = 228)

Met Syndrome cases

(n = 228)

Serum specimens available

(n = 546)

## Follow-up

Children satisfy the derived MetS classification;

age- (within 6 months) and sex- match to controls 1:1

(n = 456)

complete anthropometric and clinical data: (n = 586)

complete maternal GDM data: (n = 557)

Children who completed a 5-year visit

(n = 676)

Families recruited in FAMILY
(n = 857, including n=901 children -816 singletons)

MetS case selection: Children were classified as having a higher risk of MetS and referred to as cases if they had one or more of the following abnormalities: 1) z-BMI ≥75^th^ percentile and waist circumference ≥ median 2) z-BMI ≥75^th^ percentile and systolic blood pressure ≥90^th^ percentile 3) fasting serum glucose ≥90^th^ percentile and 4) maternal GDM diagnosis. 228 children who met these criteria were classified as “MetS cases” and were age- and sex-matched 1:1 to 228 controls.
