# Supplementary material for: Metabolite profiles and the risk of metabolic syndrome in early childhood: a case-control study
Source: BMC Med. 2021 Nov 26;19:292. doi: 10.1186/s12916-021-02162-7 (PMC8616718; doi:10.1186/s12916-021-02162-7)
Supplement: Supplementary file 2 — Additional file 2. Supplemental Methods [IDEFICS MetS definitions and MSI-CE-MS data processing] [file 12916_2021_2162_MOESM2_ESM.docx]

**Additional file 2:** **Supplemental Methods**

**IDEFICS MetS risk score derivation**

Among the 456 children, we derived the continuous IDEFICS MetS risk score for all study participants based on percentile curves created for 18745 children aged 2.0 – 10.9 years of the European IDEFICS cohort as a reference system with permission from study investigators. This was done using sex-specific and age-specific (to one decimal point) distributions based on generalized additive model for location scale and shape (GAMLSS) where the specific percentile is then converted to a z-score for each clinical parameter. GAMLSS is a recently introduced statistical approach that accounts for skewness and kurtosis. The MetS score was calculated summing age and sex-specific z-scores according to the following the formula by Ahrens et al.:

IDEFICS MetS score =

z _waist circumference_ + (z _systolic blood pressure_ + z _diastolic blood pressure_)/2 + (z _triglycerides_ – z _HDL_)/2 + z _fasting glucose_

For both systolic and diastolic blood pressure, distributions are also height-specific in addition to sex and age and for 5 children of the FAMILY cohort, whose heights were not available in the IDEFICS distribution data, the nearest available height data was used for each age category. The mean of the z-scores of both diastolic and systolic blood pressure was calculated. For the hyperglycemia component, we used glucose z-scores in the equation after conversion of fasting glucose concentrations from mmol\L to mg/dl in order to compute the z-scores from the IDEFICS distribution. For dyslipidemia the mean of the z-scores for triglycerides and HDL-C (multiplied by − 1, due to its inverse relation to MetS and converted to mg/dl) was computed. All four major cardiovascular risk factors receive the same weight in the score, as there is no clear indication that one component has a stronger influence than the rest. A higher MetS score indicates a higher cardiometabolic risk while a negative score indicates lower risk. The mean score was +0.05 and ranged from -6.1 to +6.8.

**IDEFICS MetS definition**

According to the IDEFICS criteria, MetS definition is met when the value of *three or more* risk factors (i.e. z-scores) exceeds the 90^th^ percentile for waist circumference, systolic or diastolic blood pressure, HOMA-insulin resistance or fasting glucose, and triglycerides or is lower than the 10^th^ percentile for HDL cholesterol. As per these criteria and using the externally standardized z-scores of all four risk factors in the study cohort, prevalence of MetS at the monitoring level was found to be 2.9%.

**MSI-CE-MS Data Processing**

MSI-CE-MS data was analyzed using Agilent Mass Hunter Workstation software (Qualitative Analysis, version B.06.00, Agilent Technologies, 2012). Comprehensive detection of molecular features from the raw data was performed using Mass-Hunter Molecular Feature Extractor, Molecular Formula Generator tools, and an in-house compound database. All ions were annotated using their accurate mass (m/z), relative migration time (RMT) normalized to an internal standard (Cl-Tyr, or ^13^C-glucose), and ionization mode of detection. RMTs are reported since they are an important parameter used to exclude redundant adducts and/or fragment ion peaks, which exhibit identical RMTs as the parent compound. Peak areas and migration times for all molecular features and internal standards were transferred to an Excel worksheet (Microsoft Office, Redmond, WA, USA) and RPA for each unique molecular feature were calculated. An iterative data workflow was used to reject spurious signals, redundant peaks, and background ions when performing nontargeted metabolomics based on analysis of a pooled serum sample. Principal component analysis was used for data visualization (i.e., data trends/outlier detection) when comparing the technical variance of QC samples as compared to the overall biological variance of study samples using MetaboAnalyst 4.0.
