# Supplementary material for: Metabolite profiles and the risk of metabolic syndrome in early childhood: a case-control study
Source: BMC Med. 2021 Nov 26;19:292. doi: 10.1186/s12916-021-02162-7 (PMC8616718; doi:10.1186/s12916-021-02162-7)
Supplement: Supplementary file 3 — Additional file 3: Table S1. [Serum metabolites characteristics] [file 12916_2021_2162_MOESM3_ESM.docx]

| **Additional file 3: Table S1.** Summary of 58 serum metabolites detected in FAMILY five-year old children that are annotated based on their metabolite ID, molecular formula, accurate mass (*m/z*), relative migration time (RMT), ionization mode (P = positive mode, N = negative mode), mean (SD) serum concentration (*mean relative peak area (RPA) in case of unknowns), technical precision of repeated QCs analyzed in each run, and % data completeness. | | | | | | |
| --- | --- | --- | --- | --- | --- | --- |
| # | **Metabolite ID** | **Molecular Formula** | **m/z:RMT:Ion mode** | **Mean concentration**  **(**µM**) (SD)** | **QC %CV** | **Complete Data (%)** |
| 1 | Glycine | C₂H₅NO₂ | 76.0393:0.55:P | 236.22 (59.74) | 14.6 | 100 |
| 2 | Trimethylamine-N-oxide | C_3_H_9_NO | 76.0766:0.29:P | 5.48 (3.57) | 11.8 | 99.8 |
| 3 | Alanine | C_3_H_7_NO_2_ | 90.0557:0.64:P | 235.01 (64.53) | 9.9 | 100 |
| 4 | Dimethylglycine | C_4_H_9_NO_2_ | 104.0711:0.71:P | 7.47 (2.3) | 12.1 | 100 |
| 5 | Choline | C_5_H_14_NO+ | 104.1075:0.37:P | 20.92 (7.71) | 31.7 | 100 |
| 6 | Serine | C_3_H_7_NO_3_ | 106.0500:0.77:P | 184.21 (34.33) | 6.3 | 100 |
| 7 | Creatinine | C_4_H_7_N_3_O | 114.0662:0.41:P | 21.4 (6.4) | 25.5 | 100 |
| 8 | Proline | C₅H₉NO₂ | 116.0705:0.86:P | 87 (26.92) | 6.1 | 100 |
| 9 | Valine | C_5_H_11_NO_2_ | 118.0862:0.76:P | 152 (34.23) | 16.0 | 85.7 |
| 10 | Betaine | C_5_H_12_NO_2_ | 177.1603:0.92:P | 6.52 (1.61) | 16.8 | 100 |
| 11 | Threonine | C_4_H_9_NO_3_ | 120.0654:0.84:P | 100.45 (23.87) | 6.3 | 100 |
| 12 | Unknown 1 | - | 129.0656:0.61:P | 0.11 (0.03)* | 15.1 | 100 |
| 13 | Hydroxyproline | C_5_H_9_NO_3_ | 132.0655:0.75:P | 21.59 (7.09) | 8.4 | 97.8 |
| 14 | Creatine | C_4_H_9_N_3_O_2_ | 132.0766:0.62:P | 33.68 (8.19) | 10.2 | 100 |
| 15 | Isoleucine | C_6_H_13_NO_2_ | 132.1017:0.78:P | 39.52 (8.93) | 8.6 | 100 |
| 16 | Leucine | C_6_H_13_NO_2_ | 132.1018:0.79:P | 57.33 (10.28) | 6.9 | 100 |
| 17 | Asparagine | C_4_H_8_N_2_O_3_ | 133.0573:0.84:P | 55.62 (9.28) | 8.6 | 100 |
| 18 | Ornithine | C_5_H_12_N_2_O_2_ | 133.0969:0.34:P | 54.78 (18.95) | 16.2 | 100 |
| 19 | Aspartic acid | C_4_H_7_NO_4_ | 134.0444:0.93:P | 36.38 (7.19) | 10.8 | 100 |
| 20 | Methylcysteine | C_4_H_9_NO_2_S | 136.0427:0.95:P | 14.73 (2.22) | 13.6 | 100 |
| 21 | Hypoxanthine | C_5_H_4_N_4_O | 137.0459:1.12:P | 9.67 (4.29) | 7.9 | 100 |
| 22 | Unknown 2 | C_7_H_9_N_2_O | 138.0788:0.92:P | 0.17 (0.01)* | 8.8 | 100 |
| 23 | Proline betaine | C_7_H_13_NO_2_ | 144.0988:0.91:P | 7.65 (9.56) | 16.1 | 96.9 |
| 24 | Deoxycarnitine | C_7_H_15_NO_2_ | 146.1282:0.53:P | 0.42 (0.11) | 22.2 | 100 |
| 25 | Glutamine | C_5_H_10_N_2_O_3_ | 147.0761:0.87:P | 518.2 (73.03) | 5.6 | 100 |
| 26 | Lysine | C_6_H_14_N_2_O_2_ | 147.1128:0.34:P | 123.82 (32.49) | 15.3 | 100 |
| 27 | Glutamic acid | C_5_H_9_NO_4_ | 148.0603:0.89:P | 36.35 (12.31) | 6.9 | 100 |
| 28 | Methionine | C_5_H_11_NO_2_S | 150.0583:0.85:P | 16.55 (3.23) | 5.7 | 100 |
| 29 | Histidine | C_6_H_9_N_3_O_2_ | 156.0766:0.41:P | 58.89 (11.97) | 16.3 | 100 |
| 30 | Aminooctanoic acid | C_8_H_17_NO_2_ | 160.1332:0.57:P | 12.41 (2.89) | 17.0 | 100 |
| 31 | Unknown 3 | - | 161.1281:0.37:P | 0.12 (0.08)* | 16.3 | 99.8 |
| 32 | Carnitine | C_7_H_15_NO_3_ | 162.1123:0.59:P | 15.73 (4.4) | 24.9 | 100 |
| 33 | Phenylalanine | C_9_H_11_NO_2_ | 166.0860:0.89:P | 49.85 (6.95) | 5.6 | 100 |
| 34 | Methylhistidine | C₇H₁₁N₃O₂ | 170.0922:0.44:P | 10.19 (4.57) | 12.3 | 100 |
| 35 | Arginine | C_6_H_14_N_4_O_2_ | 175.1191:0.38:P | 60.14 (13.62) | 15.3 | 100 |
| 36 | Citrulline | C_6_H_13_N_3_O_3_ | 176.1025:0.91:P | 32.42 (6.91) | 6.5 | 100 |
| 37 | Tyrosine | C_9_H_11_NO_3_ | 182.0810:0.94:P | 43.99 (7.96) | 7.1 | 100 |
| 38 | Methylarginine | C_7_H_16_N_4_O_2_ | 189.1337:0.39:P | 0.89 (0.34) | 22.2 | 99.1 |
| 39 | Asymmetric dimethylarginine | C_8_H_18_N_4_O_2_ | 203.1499:0.46:P | 1.65 (0.34) | 24.8 | 100 |
| 40 | Symmetric dimetylarginine | C_8_H_18_N_4_O_2_ | 203.1499:0.47:P | 3.74 (0.27) | 20.9 | 100 |
| 41 | Acetylcarnitine | C_9_H_17_NO_4_ | 204.1233:0.66:P | 8.17 (3.82) | 26.8 | 100 |
| 42 | Tryptophan | C_11_H_12_N_2_O_2_ | 205.0966:0.90:P | 14.33 (3.07) | 8.7 | 100 |
| 43 | Propionylcarnitine | C_10_H_19_NO_4_ | 218.1135:0.68:P | 3.48 (0.58) | 6.8 | 100 |
| 44 | Cystine | C_6_H_12_N_2_O_4_S_2_ | 241.0299:0.90:P | 36.36 (7.55) | 6.8 | 100 |
| 45 | Unknown 4 | - | 276.1191:1.14:P | 0.1 (0.02)* | 6.9 | 100 |
| 46 | Cysteinylglycine disulfide | C_8_H_15_N_3_O_5_S_2_ | 298.0526:0.72:P | 14.15 (2.73) | 8.2 | 100 |
| 47 | Lactic acid | C_3_H_6_O_3_ | 89.0252:1.93:N | 1164.18 (345.74) | 17.4 | 100 |
| 48 | 2-/3-Hydroxybutyric acids | C_4_H_8_O_3_ | 103.0400:1.65:N | 134.61 (167.79) | 40.1 | 96.7 |
| 49 | a-Ketoisovaleric acid | C_5_H_8_O_3_ | 115.0401:1.80:N | 12.63 (5.45) | 23.3 | 100 |
| 50 | Oxoproline | C_5_H_7_NO_3_ | 128.0353:1.64:N | 14.77 (6.94) | 17.7 | 100 |
| 51 | Unknown 5 | - | 128.0470:1.64:N | 0.19 (0.07)* | 20.4 | 100 |
| 52 | 3-Methyl-2-oxovaleric acid | C_6_H_10_O_3_ | 129.0557:1.65:N | 20.68 (8.78) | 19.5 | 100 |
| 53 | Xanthine | C_5_H_4_N_4_O_2_ | 151.0261:1.44:N | 5.47 (1.84) | 26.7 | 99.5 |
| 54 | Uric acid | C_5_H_4_N_4_O_3_ | 167.0210:1.51:N | 293.91 (101.35) | 20.5 | 100 |
| 55 | Hippuric acid | C_9_H_9_NO_3_ | 178.0510:1.39:N | 35.44 (35.52) | 28.6 | 91.0 |
| 56 | Glucose | C₆H₁₂O₆ | 179.0562:0.58:N | 5308.36 (405.58) | 10.6 | 100 |
| 57 | Unknown 6 | - | 248.0711:0.58:N | 0.83 (0.23)* | 34.0 | 100 |
| 58 | Phenylacetylglutamine | C_13_H_16_N_2_O_4_ | 263.1037:0.63:N | 15.66 (8.06) | 40.4 | 99.6 |
