# Supplementary material for: Metabolite profiles and the risk of metabolic syndrome in early childhood: a case-control study
Source: BMC Med. 2021 Nov 26;19:292. doi: 10.1186/s12916-021-02162-7 (PMC8616718; doi:10.1186/s12916-021-02162-7)
Supplement: Supplementary file 4 — Additional file 4: Table S2. [Unadjusted and intermediate adjusted models] [file 12916_2021_2162_MOESM4_ESM.docx]

|  | | | | | | | | | | | | |
| --- | --- | --- | --- | --- | --- | --- | --- | --- | --- | --- | --- | --- |
| **Additional file 4: Table S2:** Relation of serum metabolome profile to risk of MetS in young children with intermediate adjustments | | | | | | | | | | | | |
|  | Unadjusted | | | Model 1 | | | Model 2 | | | Model 3 | | |
| **Metabolite** | **OR** | **95% CI** | ***p-*** | **OR** | **95% CI** | ***p-*** | **OR** | **95% CI** | ***p-*** | **OR** | **95% CI** | ***p-*** |
| Glucose | 1.5 | (1.23-1.86) | 0.003 | 1.47 | (1.20-1.82) | 0.0003 | 1.51 | (1.23-1.88) | 0.0001 | 1.55 | (1.25-1.93) | 0.000079 |
| Alanine | 1.34 | (1.11-1.62) | 1.0E-04 | 1.32 | (1.09-1.60) | 0.005 | 1.32 | (1.10-1.62) | 0.004 | 1.41 | (1.16-1.73) | 0.0008 |
| Tyrosine | 1.29 | (1.07-1.57) | 0.007 | 1.28 | (1.06-1.55) | 0.012 | 1.29 | (1.07-1.57) | 0.01 | 1.33 | (1.10-1.63) | 0.004 |
| Monomethylarginine | 1.24 | (1.03-1.51) | 0.016 | 1.27 | (1.05-1.56) | 0.016 | 1.26 | (1.04-1.54) | 0.021 | 1.33 | (1.09-1.64) | 0.007 |
| Tryptophan | 0.79 | (0.66-0.96) | 0.024 | 0.80 | (0.66-0.97) | 0.023 | 0.79 | (0.65-0.96) | 0.017 | 0.78 | (0.64-0.95) | 0.015 |
| Unknown 248.0711 | 0.82 | (0.68-0.99) | 0.043 | 0.82 | (0.67-0.10) | 0.047 | 0.82 | (0.67-0.99) | 0.047 | 0.83 | (0.68-1.00) | 0.049 |
| Glutamine/Glutamate | 0.83 | (0.68-1) | 0.046 | 0.81 | (0.67-0.98) | 0.03 | 0.82 | (0.67-0.99) | 0.041 | 0.82 | (0.67-1.00) | 0.053 |
| Deoxy carnitine | 1.2 | (0.99-1.45) | 0.06 | 1.2 | (0.99-1.45) | 0.06 | 1.2 | (0.99-1.45) | 0.06 | 1.24 | (1.02-1.51) | 0.03 |
| Glutamic acid | 1.19 | (0.99-1.44) | 0.06 | 1.21 | (1.00-1.47) | 0.047 | 1.20 | (0.99-1.45) | 0.07 | 1.20 | (0.99-1.46) | 0.07 |
| Carnitine | 1.19 | (0.99-1.44) | 0.06 | 1.18 | (0.98-1.43) | 0.08 | 1.19 | (0.99-1.44) | 0.07 | 1.24 | (1.02-1.51) | 0.032 |
| Choline | 0.84 | (0.69-1.01) | 0.07 | 0.84 | (0.69-1.01) | 0.07 | 0.81 | (0.66-0.98) | 0.032 | 0.79 | (0.64-0.96) | 0.021 |
| Acetylcarnitine | 0.84 | (0.7-1.01) | 0.07 | 0.86 | (0.7-1.04) | 0.12 | 0.86 | (0.71-1.04) | 0.12 | 0.84 | (0.69-1.02) | 0.08 |
| Arginine | 1.18 | (0.98-1.43) | 0.08 | 1.16 | (0.96-1.40) | 0.13 | 1.17 | (0.97-1.42) | 0.10 | 1.21 | (1.00-1.48) | 0.052 |
| Threonine | 1.17 | (0.97-1.41) | 0.09 | 1.16 | (0.97-1.41) | 0.11 | 1.17 | (0.97-1.42) | 0.10 | 1.24 | (1.02-1.51) | 0.033 |
| Note: OR = odds ratio, CI = confidence intervals, p- = p-value for statistical significance  Model 1 adjusted for screen time exposure (6 (1.3%) missing values; final n numbers: 223 controls and 227 cases)  Model 2: adjusted for screen time exposure, diet quality score (11 (2.4%) missing values; final n numbers: 223 controls and 222 cases)  Model 3: adjusted for screen time exposure, diet quality score, physical activity, sleep time, maternal social disadvantage index (23 (5.0%) missing values; final n numbers: 216 controls and 217 cases) | | | | | | | | | | | | |
