# Supplementary material for: Metabolite profiles and the risk of metabolic syndrome in early childhood: a case-control study
Source: BMC Med. 2021 Nov 26;19:292. doi: 10.1186/s12916-021-02162-7 (PMC8616718; doi:10.1186/s12916-021-02162-7)
Supplement: Supplementary file 5 — Additional file 5: Table S3. [Pathway analysis] [file 12916_2021_2162_MOESM5_ESM.docx]

| **Additional file 5: Table S3.** Pathway analysis of metabolites associated with the MetS risk score from the multivariable linear regression. | | |
| --- | --- | --- |
| **Metabolite** | **Biochemical pathway (MetaboAnalyst)** | **Biological process related to MetS** |
| Glucose* | Glycolysis/gluconeogenesis (1/26)^#^ | Gluconeogenesis |
| Alanine* | Alanine, aspartate and glutamate metabolism (2/28)  Seleno-compound metabolism (1/20) | Gluconeogenesis  Alanine-glucose (Cahill) cycle |
| Tyrosine* | Phenylalanine, tyrosine and tryptophan biosynthesis (1/4)  Ubiquinone and other terpenoid-quinone biosynthesis (1/9)  Phenylalanine metabolism (1/10)  Tyrosine metabolism (1/42) | Aromatic amino acids metabolism |
| Threonine* | Valine, leucine and isoleucine biosynthesis (2/8)  Glycine, serine and threonine metabolism (1/33) | Amino acids metabolism |
| Carnitine* |  | Fatty acid oxidation |
| Tryptophan* | Tryptophan metabolism (1/42) | Tryptophan metabolism  Low-grade inflammation |
| Acetylcarnitine |  | Fatty acid oxidation |
| Hydroxybutyric acids | Propanoate metabolism (1/23) |  |
| Methionine | Cysteine and methionine metabolism (1/33) |  |
| Proline | Arginine and proline metabolism (3/38) |  |
| Arginine | Arginine biosynthesis (2/14)  Arginine and proline metabolism (3/38) |  |
| Monomethylarginine* |  |  |
| Glutamic acid | Arginine biosynthesis (2/14)  Arginine and proline metabolism (3/38)  Alanine, aspartate and glutamate metabolism (2/28)  Nitrogen metabolism (1/6)  D-Glutamine and D-glutamate metabolism (1/6)  Butanoate metabolism (1/15)  Histidine metabolism (1/16)  Glutathione metabolism (1/28)  Porphyrin metabolism (1/30)  Glyoxylate and dicarboxylate metabolism (1/32) | Gluconeogenesis  Glutathione metabolism |
| Unknown (m/z 129.066) |  |  |
| Glutamine/Glutamate* |  | Gluconeogenesis |
| Lysine | Lysine degradation (2/25)  Biotin metabolism (1/10) | Precursor to carnitine synthesis |
| 3-methyl-2-oxovaleric acid | Valine, leucine and isoleucine biosynthesis (2/8)  Valine, leucine and isoleucine degradation (1/40) | Amino acids metabolism |

All possible biochemical pathways were determined by MetaboAnalyst pathway analysis tool in addition to a literature search identifying biological processes relevant to MetS and related conditions.

*Consistent with adjusted logistic regression analysis

^#^ (No. of significant metabolites/total no. of metabolites involved in the pathway)
