# Supplementary material for: Metabolite profiles and the risk of metabolic syndrome in early childhood: a case-control study
Source: BMC Med. 2021 Nov 26;19:292. doi: 10.1186/s12916-021-02162-7 (PMC8616718; doi:10.1186/s12916-021-02162-7)
Supplement: Supplementary file 6 — Additional file 6: Fig. S2. [Clustering of MetS cases] [file 12916_2021_2162_MOESM6_ESM.docx]

**Additional file 6: Figure S2:** **Venn Diagram of the overlap of cardiometabolic risk factors in MetS cases**


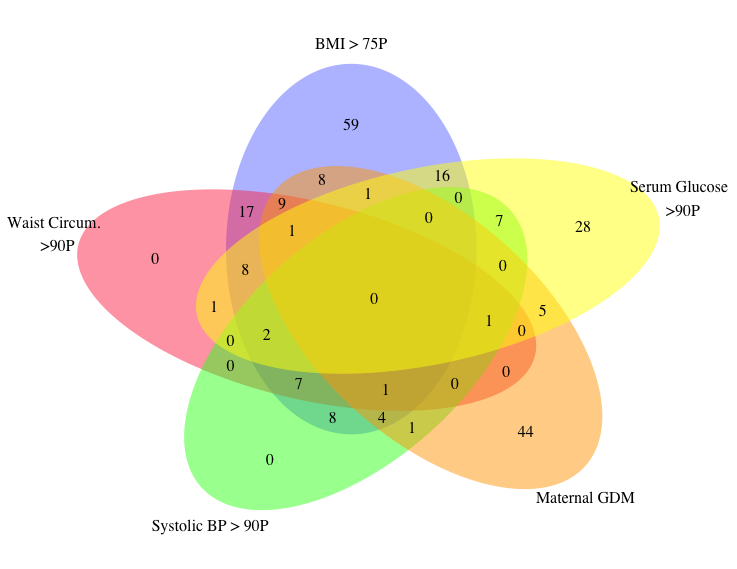


Overview of MetS risk factors and their clustering in MetS cases: Figure depicts a Venn Diagram of the overlap of risk factors in the MetS cases. None of the cases had all 5 risk factors, 34 cases had a cluster of 3 or 4 factors whereas considerable numbers of cases had a z-BMI above the 75^th^ percentile (AND waist circumference above median) or maternal GDM as the sole risk factor.
